# *Clostridium difficile* in Inflammatory Bowel Disease: A Retrospective Study

**DOI:** 10.1155/2017/4803262

**Published:** 2017-10-04

**Authors:** William Gillespie, Neil Marya, Julien Fahed, Gregory Leslie, Krunal Patel, David R. Cave

**Affiliations:** ^1^Department of Medicine, University of Massachusetts School of Medicine, 55 Lake Avenue North, Worcester, MA 01604, USA; ^2^Department of Gastroenterology, University of California Los Angeles, Los Angeles, CA, USA; ^3^Department of Gastroenterology, Aurora Healthcare, Milwaukee, WI, USA; ^4^Department of Medicine, University of Massachusetts, Worcester, MA, USA; ^5^Department of Gastroenterology, University of Massachusetts, Worcester, MA, USA

## Abstract

**Aim:**

To investigate the epidemiology and risk factors of *Clostridium difficile* infections (CDI) in patients with inflammatory bowel disease (IBD).

**Methods:**

This is a retrospective study of patients diagnosed with IBD. 1006 charts were screened and 654 patients met the inclusion criteria. Patients were divided into 2 cohorts based on the presence of prior diagnosis of CDI. Statistical analysis with Pearson's chi-squared and two-sample *t*-test was performed.

**Results:**

The incidence of CDI among IBD patients was 6.7%. There was equal prevalence of CDI among Crohn's disease (CD) (*n* = 21, 49%) and ulcerative colitis (UC) (*n* = 22, 51%). IBD patients acquired CDI at a mean age of 42.7 years, with 56% of infections acquired in the community and only 28% associated with healthcare. Only 30% of IBD patients with CDI had prior antibiotic use, and 16% had prior steroid use. IBD patients were significantly more likely to require biologic therapy (57% versus 37%, *p* < 0.01) and have extraintestinal manifestations of IBD (43% versus 28%, *p* < 0.02).

**Conclusions:**

IBD patients are more susceptible to CDI at a younger age and often lack traditional risk factors. IBD patients with at least one CDI were more likely to require biologic therapy and had greater rates of extraintestinal manifestations.

## 1. Introduction

*Clostridium difficile* is the major cause of infectious diarrhea in hospitalized patients [1] and is the primary infectious cause of pseudomembranous colitis [2]. Recent studies have shown that infections with *Clostridium difficile* (CDI) have shown increased incidence, severity, and recurrence rates over the past 10 years [3]. In addition, such infection has recently surpassed that of methicillin-resistant *Staphylococcus aureus* as the leading hospital-acquired infection in the United States [4, 5]. These infections lead to longer hospital stays, more frequent readmissions, and increased mortality among hospitalized patients [4]. Healthcare costs related to CDI are estimated to be around $1.8 billion annually in the United States, can add over $11,000 to a patient's hospital stay, and increase the length of stay by an average of 3.3 days [6]. There are several risk factors for CDI, with antibiotic use being the most well-known. Others include advanced age, increased severity of underlying illness, prior hospitalization, feeding tube use, gastrointestinal surgery, and potentially proton-pump inhibitors [7].

Patients with inflammatory bowel disease (IBD), which includes Crohn's disease (CD) and ulcerative colitis (UC), have been shown to have increased CDI rates and disproportionately higher morbidity and mortality compared to CDI patients without IBD [8, 9]. Classical risk factors may not be found in many IBD patients, and clinical findings such as pseudomembranes may not be present [3]. In contrast to archetypal epidemiological studies, CDI is now being increasingly recognized as a cause of diarrhea in the community, especially in younger individuals and in populations lacking the traditional risk factors [10–12]. This study seeks to further characterize the epidemiology and risk factors for *C. difficile* infections in IBD patients.

## 2. Methods

This was a retrospective study. The medical records of 1006 patients who carried the ICD 9 code for IBD (ICD-9: 555.x and 556.x) at a single large academic medical center from October 1, 2014, to September 30, 2015, were reviewed. Approval was obtained from the Committee for the Protection of Human Subjects for the finalized study protocol. Eligible patients were identified through a search of the electronic medical record, and data was deidentified and randomized prior to analysis. Eligible patients had a previous diagnosis of IBD based on ICD coding along with appropriate clinical symptoms and compatible endoscopic or imaging findings. 352 patients were excluded due to insufficient data to confirm IBD diagnosis or inappropriate coding. For the remaining 654 patients, extensive background epidemiologic and comorbid data were collected for each subject and incorporated into the final analysis. The data extracted from the charts included the current age of the patients, the age of IBD diagnosis, the type of IBD, peak therapy between the time of diagnosis and the current time of the study, extraintestinal manifestations, hospitalization data, inflammatory markers, and antibiotic and steroid use, among many others (Tables 1–3). For subanalysis, patients were divided into those with prior documented CDI and those without documented CDI. Statistical analysis with Pearson's chi-squared test and two-sample *t*-test was performed.

## 3. Results

### 3.1. Epidemiology

A total of 654 IBD patients were studied. 43 patients (6.7%) had at least one confirmed CDI. A review of baseline characteristics revealed a mean patient age of 42.7 years at the time of the first CDI, with 55.8% male predominance (Tables 1 and 3). This gender distribution is similar to that of IBD patients without prior CDI (52.5% male). Patients with prior CDI were more likely to have been hospitalized with at least one IBD-related admission compared to IBD patients without CDI (72% versus 42%, *p* < 0.001). The mean number of IBD-related hospitalizations of patients with CDI was 2.9, and that of IBD patients without CDI was 1.12 (*p* < 0.001). 43% of IBD patients with past CDI eventually required surgery, while only 33.5% of those without prior CDI ultimately required a surgical procedure (but this was not statistically significant with *p* = 0.15).

### 3.2. CDI Characteristics

The mean age of diagnosis of CDI in IBD patients was 42.7 years (Table 3). Interestingly, 56% of these patients had community-acquired CDI (as per the most recent IDSA guidelines [13]), with only 28% of CDI being hospital-acquired and 16% being indeterminate. Of those patients with CDI, only 30% used an antibiotic within 1 month of diagnosis. Additionally, only 16% used steroids within 1 month prior to CDI diagnosis. For the initial treatment of CDI, 44% received oral metronidazole, 32.5% received oral vancomycin, and 11% received intravenous solumedrol. Seven patients (16%) had more than one CDI (occurring >8 weeks after completion of treatment and resolution of symptoms), and two patients had multiple recurrences. Of the patients with recurrent disease, all had initial treatment with PO metronidazole. The average time to disease clearance (defined as resolution of symptoms after completion of treatment or documented negative PCR) was 20.1 days.

### 3.3. IBD Characteristics

IBD patients with at least one CDI were significantly more likely to require escalation to biologic therapy when compared to IBD patients without prior CDI (57% versus 38%, *p* < 0.01, Table 2). Only 14 patients were actively on a biologic at the time of their first CDI diagnosis. Of a total of 256 patients on biologic agents, only 14 patients (5.5% of all biologic users) developed CDI while on a biologic. There were no significant differences among the types of biologic. Only 18% of patients with prior CDI were able to control their IBD with salicylates alone, while 31% of those without prior CDI attained adequate control with salicylates, but this was not statistically significantly different (*p* = 0.08).

IBD patients with prior CDI had a higher rate of extraintestinal manifestations of IBD than those without prior CDI (43% versus 28%, *p* = 0.02). These included inflammatory arthritis, osteoporosis, osteomalacia, pyoderma gangrenosum, psoriasis, and chronic pancreatitis. The lowest mean albumin of those with prior CDI was 2.98, compared to 3.45 in those without prior CDI (*p* < 0.001). Mean peak ESR and CRP were also more elevated in those with CDI, but this was not statistically significant (*p* = 0.07 and *p* = 0.06, resp.).

With regard to the IBD subtype in patients with prior CDI, 51% had UC and 49% had CD. This distribution was similar to that of IBD patients without CDI (43% UC and 57% CD). These results show no statistically significant correlation between the IBD subtype and CDI. Overall, 8.3% of all UC patients and 6% of all CD patients studied had at least one CDI. As expected, the anatomical distribution of IBD in those with CDI predominantly involved the colon. Of those with CDI in UC, 45% had pancolitis, 31.8% had left-sided colitis, 9% had proctosigmoiditis, and 13% had proctitis. These results very closely mirror the distribution of UC in those without CDI. Similarly, in patients with CDI in CD, 23% had either isolated ileal or colonic disease, 48% had ileocolonic disease, and only 4% had isolated upper disease. This distribution is almost identical to the prevalence of CD subtypes in patients without CDI.

## 4. Discussion

Our study analyzed a large (*n* = 654) cohort of IBD patients for epidemiologic and comorbid conditions. Of these IBD patients, 43 (6.7%) had proven CDI. This incidence is concordant with that of other epidemiological studies of CDI in IBD, with rates ranging from 3% to 6% [3, 14, 15]. This rate has nearly doubled over the past decade and is far greater than that in those patients without IBD (0.04% to 0.08%) [14]. The majority of CDI in this study were diagnosed using PCR for the *C. difficile* toxin B gene in patients with clinical symptoms of infection. Two patients were diagnosed with CDI using an immunoassay for toxins A and B.

The mean age for patients with IBD who contracted CDI was only 42.7 years in this study. This is in stark contrast to the mean age of non-IBD patients most susceptible to CDI, which ranges from 65 to 75 years of age depending on the study [16–20]. Increased age is a well-recognized risk factor for CDI; however, some studies have shown that IBD patients appear to be more susceptible at a younger age [12, 15]. It is tempting to attribute this skewed age distribution to increased hospitalizations, antibiotic use, and/or immunosuppression in younger IBD patients, but these factors do not account for the majority of CDI cases in this study.

Our data shows that those IBD patients with prior CDI were significantly more likely to have had at least one hospitalization for an IBD-related cause (72% versus 41%). Additionally, those IBD patients with prior CDI were significantly more likely to have multiple hospitalizations (mean 2.9 versus 1.1). Despite the increased incidence and number of hospitalizations in those with prior CDI, our data shows that 56% of infections were community-acquired. This is in contrast to the data from Europe, Canada, and the United States suggesting a rate of 20–27% community-acquired CDI in the general population [21–23].

In this study, we used the most recent Infectious Diseases Society of America recommendations, which state that CDI is community-acquired if the patient has not been discharged from a healthcare facility in the previous 12 weeks and symptoms begin in the community or within 48 hours of admission to a hospital. Hospital-acquired CDI is defined as a symptom onset more than 48 hrs after admission or less than 4 weeks after discharge, and CDI is considered indeterminate if symptoms occur in the community between 4 and 12 weeks after discharge [14]. In contrast to the 56% rate of community-acquired CDI, only 28% of infections were hospital-acquired and 16% indeterminate. This is directly a counter to the traditional view of CDI as a predominantly healthcare-associated infection. Our results are consistent with those of other retrospective population studies of both adults and children showing that IBD patients with CDI were younger and more often had acquired infections as outpatients compared with patients without IBD [15, 24]. The majority of CDI were community-acquired; only 30% had antibiotic use within one month of diagnosis. These findings demonstrate the lack of traditional risk factors for CDI in patients with IBD. This awareness is crucial for accurate diagnosis. CDI may mimic or precipitate an IBD flare, but these two entities have distinctly divergent treatment plans. Empiric treatment with glucocorticoids in CDI without appropriate antibiotic coverage has been associated with higher short-term mortality [25], so proper diagnosis is essential.

In regard to immunosuppression, those IBD patients with at least one CDI were significantly more likely to require escalation to biologic therapy when compared to IBD patients without CDI (57% versus 38%). The overwhelming majority of biologic agents observed in this study were the TNF-alpha inhibitors infliximab and adalimumab. Similarly, only 18% of patients with prior CDI were able to control their disease with salicylates alone, while 31% of those without CDI attained adequate control with salicylates (but this was not statistically significant with *p* = 0.08). These findings could represent an increased susceptibility to CDI in those patients with more severe or aggressive IBD. In IBD, dysfunction of regulatory T cells or antigen-presenting cells can lead to a loss of tolerance to ubiquitous microbial antigens [26]. The concentrations of mucosal bacteria of the colon have been shown to increase progressively with the severity of disease, both in the inflamed and noninflamed colon [27]. While the results of specific microbial dysbiosis in IBD vary between studies, the most consistent observation is reduced bacterial diversity [28]. It is known that reduced microbial diversity in the gut is a common pathogenic pathway for CDI; therefore, those patients with more severe IBD may be more innately susceptible to CDI.

Perhaps, a more apparent cause of increased CDI in this population would be the direct immunosuppressive effects of biologic agents themselves. However, studies have shown conflicting data on the role of TNF-alpha inhibitors such as infliximab and adalimumab in increasing susceptibility to CDI [28–30]. In our study, the majority of patients who had CDI would eventually require a biologic; however, only 14 patients were actively on a biologic at the time of CDI diagnosis. In addition, there was no recurrence of CDI after patients were started on a biologic. Overall, only 5.4% of the 256 patients on a biologic agent were diagnosed with CDI. Similarly, only 16% of patients were on systemic corticosteroids or immunomodulators within one month of CDI diagnosis. While immunosuppressive agents certainly increase susceptibility to opportunistic infections, they did not appear to be the principal risk factor for CDI in this study.

Patients with at least one CDI were more likely to have extraintestinal manifestations of IBD than those patients without CDI (43% versus 28%). These included inflammatory arthritis, osteoporosis, osteomalacia, pyoderma gangrenosum, psoriasis, and chronic pancreatitis. While both arthritis and osteoporosis are very common conditions, the majority of these patients developed these conditions at an age younger than 60 years. IBD patients with at least one CDI also had lower mean albumin than those without CDI (2.98 versus 3.45).

There are some limitations to this study. This study was retrospective. We were unable to evaluate the severity of CDI due to the large proportion of community-acquired and indeterminate infections. Data was provided by a large number of outside providers with variability in data collected or accessible around the time of diagnosis. Similarly, the CDI cohort consists of only those patients who were admitted to our hospital with CDI or were followed up by one of our outpatient providers around the time of diagnosis. There are likely patients in the IBD cohort who were diagnosed or treated for CDI at outside facilities without our knowledge, leading to underestimation of CDI incidence and an incomplete CDI cohort. Finally, the toxin B PCR cannot distinguish between new infection and asymptomatic carriage, complicating diagnosis in this population given the similarity of symptoms between CDI and an IBD flare.

This study demonstrates an equal prevalence of CDI in UC and CD. It shows that IBD patients are more susceptible to CDI at a younger age and often lack traditional risk factors such as recent hospitalization or antibiotic use. Additionally, IBD patients with at least one CDI were more likely to require biologic therapy, had lower success with salicylates, had lower mean albumin, and had greater rates of extraintestinal manifestations.

## Figures and Tables

**Table 1:** IBD demographics.

	IBD patients with prior CDI (*n* = 43)	IBD patients without prior CDI (*n* = 611)
Male, *n* (%)	24 (55.8)	321 (52.5)
Crohn's disease, *n* (%)	21 (48.8)	344 (56.3)
Ileal	5 (23.8)	89 (25.6)
Colonic	5 (23.8)	81 (23.2)
Ileocolonic	10 (47.6)	150 (43.1)
Isolated upper disease	1 (4.8)	24 (6.9)
Ulcerative colitis, *n* (%)	22 (51.2)	267 (43.6)
Pancolitis	10 (45.5)	119 (46.3)
Left-sided colitis	7 (31.8)	60 (23.3)
Proctosigmoiditis	2 (9)	50 (19.4)
Proctitis	3 (13.6)	28 (10.9)

**Table 2:** IBD characteristics.

	IBD patients with prior CDI (*n* = 43)	IBD patients without prior CDI (*n* = 611)	Univariate analysis
			*p* value
≥1 IBD-related hospitalization, *n* (%)	32 (72)	255 (41.7)	*p* < 0.001
Number of IBD-related hospitalizations, mean ± SD	2.9 ± 3.1	1.12 ± 2.27	*p* < 0.001
Surgery for IBD, *n* (%)	19 (43.2)	205 (33.5)	*p* = 0.150
Biologic as peak IBD therapy, *n* (%)	25 (56.8)	231 (37.8)	*p* = 0.008
Salicylate as peak IBD therapy, *n* (%)	8 (18)	189 (30.9)	*p* = 0.088
Extraintestinal manifestations of IBD, *n* (%)	19 (43)	174 (28.4)	*p* = 0.021
Lowest albumin, mean ± SD	2.98 ± 0.83	3.45 ± 0.79	*p* < 0.001
Peak CRP, mean ± SD	55.9 ± 64.8	37.8 ± 58.3	*p* = 0.065
Peak ESR, mean ± SD	43.5 ± 31.5	35 ± 28.2	*p* = 0.071

CRP = C-reactive protein, ESR = erythrocyte sedimentation rate.

**Table 3:** CDI characteristics.

	IBD patients with prior CDI (*n* = 43)
Age at first CDI (years), mean ± SD	42.7 ± 19.9
CDI etiology, *n* (%)	
Community-acquired	24 (56)
Hospital-acquired	12 (28)
Indeterminate	7 (16)
Antibiotic use prior to CDI, *n* (%)	13 (30)
Steroid use prior to CDI, *n* (%)	7 (16.3)
Biologic use at the time of CDI, *n* (%)	14 (32.5)
Time to CDI clearance (days), mean ± SD	20.6 ± 14.27
